# Editorial: Dietary Strategies for Healthy Aging—Caloric Restriction and Beyond

**DOI:** 10.3389/fnut.2022.866928

**Published:** 2022-03-01

**Authors:** Sebastian J. Hofer, Sergio Davinelli

**Affiliations:** ^1^Institute of Molecular Biosciences, NAWI Graz, University of Graz, Graz, Austria; ^2^Department of Medicine and Health Sciences “V. Tiberio”, University of Molise, Campobasso, Italy

**Keywords:** caloric restriction, aging, nutrition, healthspan, caloric restriction mimetic

Ample evidence generated over the past two decades underlines the crucial role of nutrition to achieve healthy aging. Numerous dietary strategies and food components have increasingly been investigated as interventions to preserve function and compress morbidity and disability to a period later in life. Findings from preclinical investigations, as well as cross-sectional, longitudinal, and intervention studies indicated that well-established ‘holistic' healthy diets [e.g., DASH (1) and Mediterranean diets (2, 3)] and specific dietary components [e.g., polyamines (4), and polyphenols (5)] may exert health-promoting effects during aging.

Among these dietary strategies, caloric restriction (CR), an ambiguous term often synonymously used with energy restriction (ER), has proven to be a very robust and broadly applicable intervention that affects aging-relevant molecular pathways and induces a range of beneficial effects involved in the maintenance of physiological functions throughout the life course (6, 7). Generally, CR is defined as a reduction in daily caloric intake without malnutrition. Different dietary alternative strategies have been developed to produce CR-like effects. Prominent examples include intermittent fasting (IF) and alternate-day fasting (ADF) (8). However, since the real-life applicability of CR is hardly sustainable in the long-term, the development of pharmacological mimetics of CR has gained considerable attention (9, 10). Multiple screenings have been performed or are currently taking place to identify synthetic or natural molecules that can be used to mimic CR phenotypes.

This Research Topic contains 11 articles covering the specific parts of the above-mentioned aspects.

Frailty is a prevalent age-related clinical condition generally recognized as a state of high vulnerability to adverse health outcomes (11). Although its etiology remains poorly understood, specific dietary regimens may prevent, delay, or even reverse this condition. Liu et al. summarize evidences on the impact of CR and alternative approaches on frailty syndrome, highlighting potential underlying mechanisms. Besides frailty, older adults are particularly vulnerable to cardiometabolic disease and unhealthy diet is a leading risk factor for the development of cardiometabolic disease (12). Perry et al. have examined the response of a caloric-restricted DASH (Dietary Approaches to Stop Hypertension) diet on parameters of cardiometabolic health in a cohort of sedentary obese older adults. They found that a DASH-like diet with restricted calories is an effective approach to improve cardiovascular, metabolic, and inflammatory biomarkers. Besides calories, macronutrients play an important role in shaping a diet's effects on health and aging (13). Dietary fiber is considered to contribute no calories to our diet (14) and its intake is associated with lower risk of type 2 diabetes, cardiovascular disease, and weight gain. However, the antihypertensive effect of dietary fiber intake has not been elucidated until now. Results from the SWAN study published in this e-collection revealed that intake of dietary fiber from grains contributes to a lower risk of hypertension in midlife women.

In this scenario, Voglhuber et al. provide an excellent overview of alternatives to CR (e.g., IF, ADF, and the Mediterranean diet) that could mimic the cardiometabolic benefits of CR in animal models and humans. The authors also summarize emerging potential CR mimetics, such as spermidine, NAD^+^ precursors, and metformin, highlighting the mechanism underpinning their cardiometabolic and health-promoting effects. Similarly, Stadler and Marsche provide an insightful overview, detailing how dietary strategies and various nutritional components may improve the atheroprotective functions of high-density lipoproteins (HDL). They discuss the clinical efficacy of Mediterranean diet, CR, and IF to increase HDL functionality and promote cardiovascular health. Furthermore, these authors address this topic by focusing on phenolic compounds that could exert positive effects on HDL function.

As briefly discussed in the articles by Voglhuber et al., as well as Stadler and Marsche, CR and fasting applications in real-life contexts harbor significant challenges. Hence, the concept of CR mimetics (CRMs) was developed to identify bioactive substances that mimic the molecular effects induced by CR (9, 10, 15). Recently, this research field has gained an increasing scientific interest. Therefore, in Hofer et al. we have performed a comprehensive literature review describing a wide range of naturally occurring substances with CR-mimicking properties, along with their dietary sources, intake levels and the current state of clinical research. Despite challenges in assimilating experimental findings into clinical treatments, CRM candidates, such as polyamines, polyphenols, NAD^+^ precursors, and glycolytic inhibitors, appear to prolong life- and healthspan in model organisms. Accordingly, the consumption of CRMs may elicit numerous disease-protecting activities, including neuroprotective effects. In line, the paper from de Vries et al. presents a systematic review and meta-analysis on the brain-enhancing effects of polyphenols, a promising source of potential CRMs. They included 66 randomized clinical trials with participants aged 40 years or older. The results indicate that short- and moderate-term supplementation with some classes of polyphenols may improve working and episodic memory in non-pathological and pathological aging.

Although religious fasting is practiced by millions of believers around the world, scarce data exist on its impact on human health. For centuries, humans have adopted various forms of religious fasting as regimens of spiritual purification. Bahá'í fasting is a specific form of intermittent dry fasting characterized by abstaining from food and liquids during daylight hours every year in March for 19 consecutive days. Koppold-Liebscher et al. conducted a prospective exploratory cohort study in Bahá'í volunteers to evaluate safety and effects of Bahá'í fasting on hydration, metabolism, and circadian clock. The study revealed that this form of fasting is safe with no negative effects on hydration biomarkers. Furthermore, Bahá'í fasting appears to improve fat metabolism and cause only transient alterations of circadian rhythms. Despite the relatively small number of studies on this topic, fasting treatments are also associated with detoxifying properties. Grundler et al. explored the effects of long-term fasting (10 days) on the excretion of heavy metals and glyphosate in 109 healthy subjects. The results of the study provide first insights into the changes in heavy metal excretion after fasting and show a reduction in the urinary levels of arsenic and nickel, as well as a reduction in hair lead levels.

The opinion article from Ostojic discusses the importance of the creatine transporter (CT1), which is located on the plasma membrane of various energy-demanding cells and whose dysfunction is associated with movement and behavior disorders. A potential but barely explored cellular effect of CR could be an increase in CT1 activity, facilitating creatinine uptake.

Finally, Wahl and LaRocca present a detailed review of the transcriptomic effects of healthy nutritional interventions, including CR, IF, ADF, prolonged fasting, time-restricted feeding, and protein and amino acid restriction. They describe gaps in the research, highlighting the importance of transcriptome/multi-omic studies to better understand the effects of these dietary interventions.

Despite the richness and importance of the topics covered in the papers published in this e-collection, the complex impact of CR and CR-alternatives on aging and health is still insufficiently understood and there are many aspects to be clarified in this fascinating research field.

## Author Contributions

Both authors listed have made a substantial, direct, and intellectual contribution to the work and approved it for publication.

## Conflict of Interest

The authors declare that the research was conducted in the absence of any commercial or financial relationships that could be construed as a potential conflict of interest.

## Publisher's Note

All claims expressed in this article are solely those of the authors and do not necessarily represent those of their affiliated organizations, or those of the publisher, the editors and the reviewers. Any product that may be evaluated in this article, or claim that may be made by its manufacturer, is not guaranteed or endorsed by the publisher.
